# Coevolution of Trustful Buyers and Cooperative Sellers in the Trust Game

**DOI:** 10.1371/journal.pone.0044169

**Published:** 2012-09-07

**Authors:** Naoki Masuda, Mitsuhiro Nakamura

**Affiliations:** 1 Department of Mathematical Informatics, The University of Tokyo, Bunkyo, Tokyo, Japan; 2 PRESTO, Japan Science and Technology Agency, Kawaguchi, Saitama, Japan; Hungarian Academy of Sciences, Hungary

## Abstract

Many online marketplaces enjoy great success. Buyers and sellers in successful markets carry out cooperative transactions even if they do not know each other in advance and a moral hazard exists. An indispensable component that enables cooperation in such social dilemma situations is the reputation system. Under the reputation system, a buyer can avoid transacting with a seller with a bad reputation. A transaction in online marketplaces is better modeled by the trust game than other social dilemma games, including the donation game and the prisoner's dilemma. In addition, most individuals participate mostly as buyers or sellers; each individual does not play the two roles with equal probability. Although the reputation mechanism is known to be able to remove the moral hazard in games with asymmetric roles, competition between different strategies and population dynamics of such a game are not sufficiently understood. On the other hand, existing models of reputation-based cooperation, also known as indirect reciprocity, are based on the symmetric donation game. We analyze the trust game with two fixed roles, where trustees (i.e., sellers) but not investors (i.e., buyers) possess reputation scores. We study the equilibria and the replicator dynamics of the game. We show that the reputation mechanism enables cooperation between unacquainted buyers and sellers under fairly generous conditions, even when such a cooperative equilibrium coexists with an asocial equilibrium in which buyers do not buy and sellers cheat. In addition, we show that not many buyers may care about the seller's reputation under cooperative equilibrium. Buyers' trusting behavior and sellers' reputation-driven cooperative behavior coevolve to alleviate the social dilemma.

## Introduction

The number of transactions executed in online marketplaces such as eBay is soaring up. To buy a desired item, a buyer must first trust in a seller by paying in advance. Because the seller may lose little by dismissing a single buyer, the seller may be tempted to ship a counterfeit item or may not even transport the purchase to the buyer. If many sellers behave maliciously toward buyers, the marketplace would collapse. Here is a moral hazard. A classical example in which counterfeit items would prevail is the “market for lemons” or the used car market [1]. Nevertheless, many auction sites and related online services, including opinion forums, price comparison sites, and product review sites, enjoy prosperity without seriously being swamped by the malicious behavior of users [2]–[5].

The main mechanism to elicit the cooperative behavior of sellers in such a social dilemma situation is the online reputation management system, also called the feedback mechanism [2]–[5]. When a reputation management system is implemented, a buyer is invited to evaluate the seller after a transaction so that other buyers can refer to the reputation of this seller in the future. A seller with a good overall reputation would successfully sell items to many buyers in the long run, whereas a seller with a bad reputation would be avoided by buyers. In a seminal paper, Klein and Leffler analyzed the role of reputations in alleviating a moral hazard [6].

Reputation mechanisms in online marketplaces are often cited as a successful example of indirect reciprocity [7], [8]. Indirect reciprocity (precisely, downstream or vicarious reciprocity as its major subtype [8], [9]) is a mechanism for the alleviation of social dilemmas. It dictates that an individual 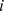 with a good reputation is helped by another individual that 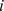 has not met. 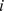 may also help other individuals that 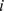 does not know. In fact, most buyer-seller pairs conduct only one transaction on eBay, such that a buyer probably does not know the seller with whom the buyer is about to transact [10]. It is theoretically established that indirect reciprocity enables cooperation in social dilemma games under proper conditions [8], [9], [11]–[16].

However, the mechanism governing cooperation between unacquainted buyers and sellers observed in real online transactions does not resemble that provided by these models. The existing models of indirect reciprocity are mostly based on the donation game, which is a type of social dilemma games. In the donation game, two players are chosen from a population, one as donor and the other as recipient. If the donor helps the recipient, the recipient gains a benefit, which is larger than the cost that the donor is charged. If the donor does not help the recipient, the donor and the recipient gain nothing. The donor's help contributes to social welfare, whereas the donor is better off withholding the help.

In the previous models of indirect reciprocity [8], [9], [11]–[22], each player is selected as many times as donor and recipient per generation (we discuss an exception [23] in the Discussion). Therefore, the players are essentially involved in a symmetric prisoner's dilemma game. In contrast, the social dilemma game effectively played in online marketplaces is a highly asymmetric game. Buyers and sellers are distinct roles that each individual does not play with equal probability [10]. In addition, in the donation game and the symmetric prisoner's dilemma, there is no concept wherein one player invests trust in a peer player in the one-shot game. Theoretical models of reputation-based cooperation using different social dilemma games such as the ultimatum game also assume symmetrization of the two roles [24], [25]. This is also the case for the models of reputation-based cooperation analyzed in economic literature [26], [27].

**Figure 1:**
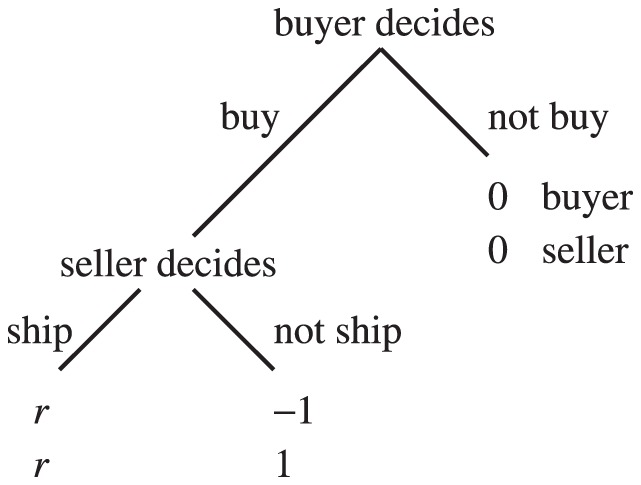
Schematic of the trust game.

The trust game [28]–[31] seems to be a much better model for online marketplaces [7]. As shown in Fig. 1, the buyer, also called the investor, first decides whether to buy an item from the seller. If the buyer buys, the seller, also called the trustee, decides whether to ship the appropriate item to the buyer. If the buyer buys and the seller does not ship, succumbing to the temptation to defect, the seller gains the largest payoff 1, and the buyer gains the smallest payoff 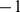. If the buyer buys and the seller ships, both players obtain a relatively large payoff 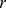, where 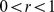. If the buyer does not buy, both players gain a relatively small payoff 0, which is the unique Nash equilibrium of the game. A cooperative society is realized when the buyer buys and the seller ships. In laboratory experiments, humans cooperate to some extent in the trust game [30]–[33], and reputation mechanisms enhance cooperation [7], [34]–[36]. The trust game with a reputation mechanism also approximates the situation of commerce conducted by the medieval Maghribi traders [37], [38] and the market for lemons (if a gossip-based reputation mechanism is operational) [1].

It was shown in a seminal paper that cooperation based on reputations is possible in the essentially asymmetric trust game [6]. Nevertheless, several important questions in this framework are theoretically unresolved. Is cooperation more ubiquitous than uncooperation, in particular when these two situations are both stable equilibria? How do different reputation-based strategies of buyers compete in a population? How does the reputation-based cooperation emerge through population dynamics? Non-evolutionary numerical results for the trust game with reputation mechanisms [39], [40] do not explain the stability and emergence of cooperation. An evolutionary theory [9], [25] and numerical simulations [41] showed that reputations induce cooperation in the trust game. However, in these papers, each player serves the two roles with equal probability such that the game is essentially the symmetric prisoner's dilemma.

We theoretically clarify the possibility of reputation-based cooperation in the trust game by analyzing the Nash equilibria and the coevolutionary replicator dynamics of buyers and sellers. We show that coevolution of cooperative buyers and sellers is realized relatively easily. In particular, the fraction of buyers that score sellers does not have to be large, and many buyers do not discriminate between good and bad sellers even when cooperation prevails.

## Model

### Trust game

We analyze equilibria and evolutionary dynamics of the trust game (Fig. 1) in an infinite population of four types of buyers and two types of sellers under a reputation mechanism. Each seller possesses the binary reputation, good (G) or bad (B), that dynamically changes as a result of the single-shot trust game. Referring to the two reputations as G and B is purely conventional.

In a time unit, each seller 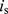 plays the trust game with a randomly chosen buyer 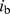. The buyer 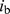 first decides whether to buy an item from 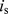 possibly on the basis of 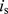
's reputation. If 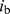 does not buy, no transaction occurs, leaving the payoff 0 to both 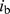 and 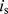. If 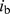 buys, 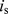 decides whether to ship the item (cooperate; C) or not (defect; D). If 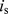 ships the item, then both 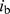 and 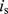 obtain 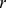. If 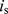 does not ship the item and exploits 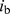, 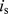 gains 1, and 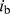 gains 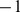. If 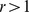, it is beneficial for both players to cooperate. If 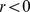, transaction would never occur irrespective of the reputation mechanism. We set 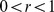 to represent the social dilemma.

We repeat the procedure explained above for 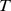 time units such that each player plays the trust game 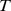 times on an average. We assume that 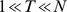 such that the probability that the same pair of buyer and seller interact more than once within time 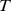 is infinitesimally small.

### Social norms

When 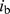 decides to buy, 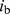 assigns G or B to 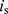 on the basis of 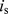
's action. The new reputation of 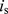 is instantaneously propagated to the entire population such that any buyer can refer to this information when playing with 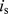 in later times. We refer to the rule according to which the buyer assigns a reputation to the seller as a social norm.

An intuitively rational social norm, which reputation mechanisms in successful online marketplaces apply, is to assign G and B when 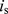 has cooperated and defected, respectively. This norm is called image scoring [11], [12]. Alternatively, 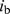 may assign G no matter whether 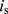 cooperates or defects. We call this social norm indifferent scoring. A buyer is assumed to commit the assignment error with probability 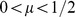 such that the seller receives a reputation that is contrary to what is expected from the social norm.

In fact, some but not all of the buyers may be interested in rating sellers [2]–[4]. To investigate this scenario, we consider a case in which a buyer has a unique scoring type as well as strategy. We assume that a fraction of 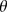 and 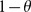 buyers are image scorers and indifferent scorers, respectively. We also assume that the scoring type does not affect the buyer's payoff, such that the fraction of image scorers in the population does not change in the course of the replicator dynamics. Alternatively, we can assume that each buyer is a permanent indifferent scorer or permanent image scorer. This assumption may be controversial. We will return to this issue in Discussion.

We do not assume the reputation for buyers because in online marketplaces, the impact of the seller's reputation is much larger than that of the buyer's reputation [3], [5]. Previous laboratory experiments that modeled the situation in auction sites also neglected the reputation of buyers [7], [10], [34].

### Strategies

The probability that the buyer decides to buy from G and B sellers is denoted by 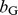 and 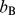, respectively. We consider four strategies for buyers: unconditional buyer (Buy) specified by 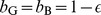, where 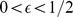; discriminator (Disc) specified by 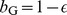, 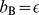; anti-discriminator (AntiDisc) specified by 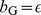, 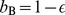; and unconditional no-buyer (NoBuy) specified by 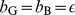.

As introduced in section “Trust game”, sellers have two strategies, C and D. For simplicity, we assume that the seller's action is deterministic, whereas the buyer's action is stochastic.

## Results

### Payoffs

The reputation of a seller obeys a Markov chain with two states G and B. To obtain the payoffs, we adopt the deterministic calculations developed by Ohtsuki and Iwasa [15], [18], [19].

If 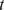 (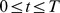) is sufficiently large, we can approximate the probability of the G reputation for a C seller and that for a D seller by the population averages denoted by 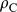 and 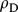, respectively. The dynamics of the reputation averaged over the C sellers and that averaged over the D sellers are represented as
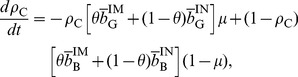
(1)

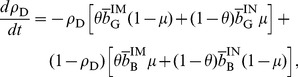
(2)where 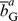 and 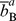 (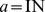 for indifferent scorer or 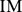 for image scorer) are the probabilities that the buyer of scoring type 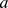 decides to buy from a G and B seller, respectively. For example, 
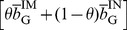
 on the right-hand side of Eq. (1) represents the probability that a buyer decides to buy from a C seller. Because the multiplicative factor 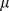 represents the probability that the C seller that has cooperated mistakenly receives B reputation, the first term on the right-hand side of Eq. (1) represents the case where the reputation of C seller turns from G to B. Similarly, the second term represents the case where the reputation of C seller turns from B to G. It should be noted that the seller's reputation does not change when the buyer decides not to buy.

The probabilities that the buyer decides to buy from a G and B seller are respectively represented as
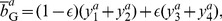
(3)

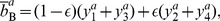
(4)where 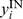 and 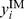 are the fractions of buyers with strategy 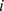 among the indifferent scorers and among the image scorers, respectively. Buy, Disc, AntiDisc, and NoBuy correspond to 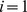, 2, 3, and 4, respectively; 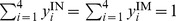.

For simplicity, we assume that
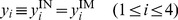
(5)is initially satisfied. In other words, the scoring type and strategy are independent of each other. Note that 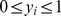 (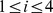) and 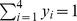. Because Eqs. (3), (4), and (5) imply 
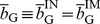
 and 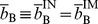, Eqs. (1) and (2) give the limit values
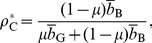
(6)

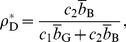
(7)where
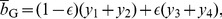
(8)

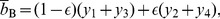
(9)and we set
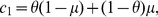
(10)

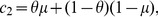
(11)for notational convenience. Equation (6) does not depend on 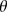, which reflects the fact that both the indifferent and image scorers evaluate C sellers as G with a large probability 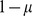 (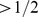). In contrast, Eq. (7) implies that 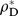 decreases with 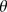. This is because the image scorer may issue a B reputation with a large probability, whereas the indifferent scorer does not.

For sufficiently large 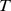, the reputations are in the equilibrium almost all the time. Then, the buyer's payoff should be equal to
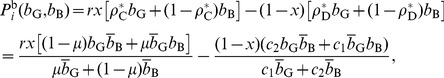
(12)where
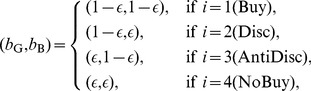
(13)and 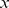 (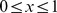) is the fraction of C sellers. The payoffs for C and D sellers are given by
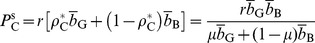
(14)and
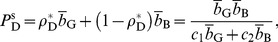
(15)respectively.

Even if we consider the stochastic dynamics of the reputation and the buyer's action, the values of the payoffs derived above give the precise mean values.

#### Proposition 1

Regardless of the initial reputation value of a seller, in the limit 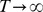 and 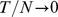, the expected payoff for the buyer is given by Eq. (12) and that for the seller is given by Eqs. (14) and (15).

### Proof of Proposition 1

The reputation score of the seller obeys a Markov chain with two states. Consider a C seller 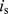 with reputation G. The G reputation does not change in one time step if the paired buyer 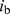 decides to buy with probability 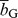 and correctly assign G to 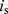 with probability 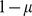 or, 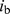 decides not to buy with probability 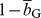. Otherwise, 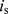
's reputation turns into B. When 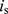 has reputation B, it is unchanged if 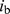 decides to buy with probability 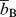 and commit assignment error with probability 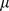, or 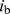 decides not to buy with probability 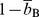. Otherwise, 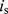
's reputation turns into G.

Therefore, the transition matrix of the Markov chain is represented as
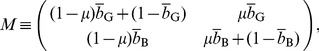
(16)where 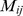 (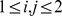) represents the transition probability from state 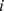 to state 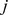, and we associate G and B with states 1 and 2, respectively. Because 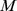 is a nondegenerate (right) stochastic matrix, we can decompose 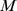 using the left and right eigenvectors corresponding to eigenvalue 1 as
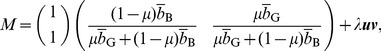
(17)where 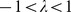 is the other eigenvalue of 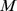, and 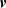 and 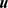 are the left and right eigenvectors corresponding to 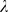, respectively. We do not calculate 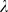, 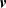, and 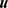 because their values are immaterial in the following arguments. Note that the eigenvectors are normalized such that
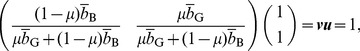
(18)

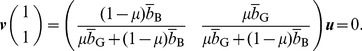
(19)


Assume that the C seller 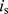 initially has reputation G and B with probability 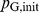 and 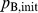, respectively, where 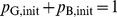. Then, the probability that 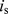
's reputation is G and B after playing 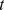 games is given by the first and second columns of 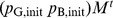, respectively.

The expected payoff for 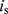 in a single game is equal to 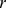 multiplied by the probability that 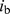 decides to buy. Therefore, the expected payoff for 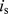 per single game, averaged over 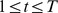, converges in the limit 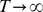 to
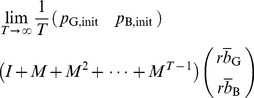
(20)

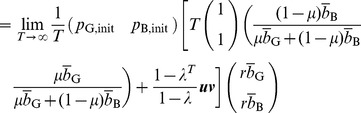
(21)

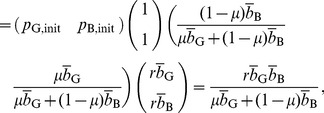
(22)which reproduces Eq. (14). The expected payoff for the D seller, given by Eq. (15), can be derived in the same manner.

Next, we calculate the payoff for a Buy buyer 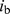. In each time step, the expected number of C seller with reputation G and B with whom 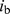 is paired is equal to the first and second columns of 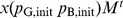, respectively. When the C seller 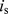 has reputation G (B), the expected payoff for 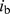 in a single game is equal to 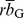 (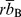). Therefore, the contribution of the C seller to the expected payoff for 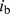 per single game, averaged over 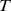 games, converges to
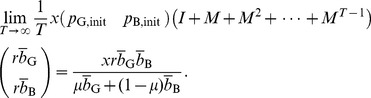
(23)This quantity is equal to the first term on the right-hand side of Eq. (12) when 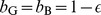 (i.e., Buy). Analogous calculations for the case of D seller yields the second term on the right-hand side of Eq. (12) when 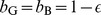. The payoff for Disc, AntiDisc, and NoBuy can be calculated in a similar manner. Here is the end of the proof.

### Nash equilibria

Based on the expected payoff determined by Proposition 1, we identify the equilibria of the game. In the analysis, we exploit the fact that 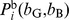 is linear in 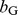 and 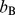. There are three types of Nash equilibria. The so-called uncooperative equilibrium is composed of NoBuy and the D seller. In the so-called cooperative equilibrium, Buy and Disc are mixed in the buyer's strategy and the probability of C is large in the seller's strategy. The cooperative equilibrium corresponds to the situation in which buyers and sellers do not repeatedly interact but trust each other on the basis of the reputation mechanism. When it exists, it coexists with the uncooperative equilibrium. The other equilibrium appears only for a singular parameter set. Therefore, we are not concerned with it in the later analysis.

#### Proposition 2

The asymmetric trust game with a reputation mechanism whose expected payoffs are defined by Eqs. (12), (14), and (15) possesses the following three types of Nash equilibria.

1. Uncooperative equilibrium: combination of NoBuy and 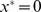. This pure-strategy Nash equilibrium is also strict.
2. Cooperative equilibrium: the mixture of Buy and Disc, with the probability of Buy and Disc being
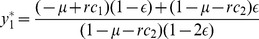
(24)and 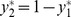, respectively. The probability of C seller is given by
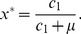
(25)The cooperative equilibrium exists when
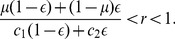
(26)The cooperative equilibrium is also asymptotically stable under the replicator dynamics. For completeness, the replicator dynamics of buyers and sellers are given by
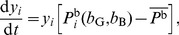
(27)

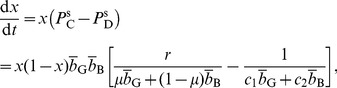
(28)respectively, where the buyer's mean payoff is given by
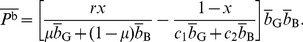
(29)
3. A singular equilibrium: combination of Disc buyer and a mixed seller's strategy with any 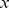 satisfying
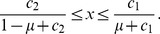
(30)This equilibrium exists when
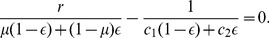
(31)

We remark that the cooperative equilibrium is called so because 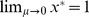. We prove Proposition 2 in the next section.

It should be noted that extending the concept of the evolutionary stability to the asymmetric game is not straightforward. In the matrix game, a strictly (i.e., completely) mixed Nash equilibrium cannot be an asymptotically stable equilibrium under the replicator dynamics, and an evolutionarily stable strategy in the asymmetric game is necessarily a (pure) strict Nash equilibrium [42]–[45]. Nevertheless, Proposition 2 dictates that the cooperative equilibrium is an asymptotically stable strictly mixed strategy. This is possible because the payoff values are density-dependent in our model; it is not a matrix game. Therefore, we directly prove that the cooperative equilibrium is asymptotically stable in the replicator dynamics.

### Proof of Proposition 2

We identify all the mixed-strategy Nash equilibria of the asymmetric game whose payoffs are given by Eqs. (12), (14), and (15).

#### One buyer's strategy

Consider a possible equilibrium composed of a single buyer's strategy. If there is only Buy, AntiDisc, or NoBuy, we substitute 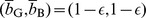, 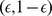, and 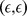, respectively, in Eqs. (14) and (15) to obtain 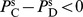 for 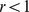. Therefore, 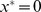 must be satisfied in a possible Nash equilibrium.

When 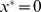, Eq. (12) is simplified to
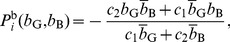
(32)where 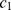 and 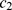 are defined in Eqs. (10) and (11), respectively. Equation (32) implies that the payoff for NoBuy is larger than those for Buy, Disc, and AntiDisc. Therefore, the combination of NoBuy and D seller is the only (strict) Nash equilibrium allowed in this regime. We call this equilibrium the uncooperative equilibrium.

Suppose instead that there is only Disc. If
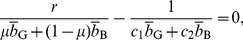
(33)where 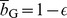 and 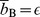, we obtain 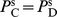 for any 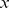. Substituting Eq. (33) in Eq. (12) yields
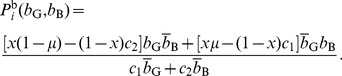
(34)
Equation (34) indicates that Disc obtains a payoff larger than or equal to those of Buy, AntiDisc, and NoBuy if
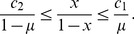
(35)The range of 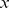 that satisfies Eq. (35) always exists because 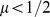 guarantees 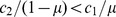. Therefore, the combination of Disc and Eq. (35), i.e.,
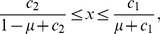
(36)yields Nash equilibria.

If Eq. (33) is not satisfied, which is a generic case, only 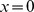 and 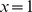 may result in a Nash equilibrium because the difference between Eqs. (14) and (15) is independent of 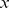. If 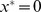 (i.e., 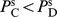), Eq. (12) is simplified to
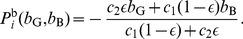
(37)
Equation (37) implies that NoBuy gains a larger payoff than Disc. If 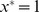, substituting 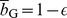 and 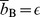 in Eqs. (14) and (15) and setting 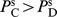 lead to the following necessary condition for Disc to be Nash:
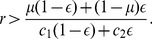
(38)
Equation (38) replicates Eq. (26). When Eq. (38) is satisfied, substituting 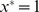, 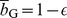, and 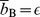 in Eq. (12) yields
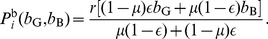
(39)Therefore, Buy gains a larger payoff than Disc, such that the pure Disc is not Nash.

In conclusion, the only pure (strict) Nash equilibrium is the uncooperative equilibrium composed of NoBuy and D seller.

#### Mixture of two buyer's strategies

Consider the mixed strategies (i.e., coexistence) of two buyer's strategies as candidates of Nash equilibria. If we select two strategies out of Buy, AntiDisc, and NoBuy, we can show 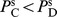 in a manner similar to the case of the one buyer's strategy. In this case, 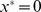 must hold true in the equilibrium. Equation (12) with 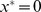 indicates that the payoff for NoBuy is larger than that for AntiDisc, which is larger than that for Buy. Therefore, such a mixed-strategy Nash equilibrium, in which the payoff for the two strategies of buyers must be the same, does not exist. This implies that Disc must be selected as one of the two buyer's strategies in a possible Nash equilibrium.

*Mixture of Buy and Disc: cooperative equilibrium*: Consider a mixture of Buy and Disc, which we call the cooperative equilibrium. Note that
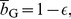
(40)

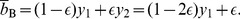
(41)Because 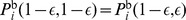 must be satisfied in the Nash equilibrium, the coefficient of 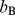 in Eq. (12) must be equal to 0. This condition combined with 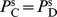 yields
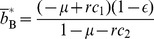
(42)and
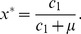
(43)
Equation (43) replicates Eq. (25).

The mixture of Buy and Disc is equivalent to 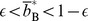. The inequality 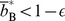 is always satisfied if 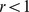 (note that the denominator of Eq. (42) is always positive if 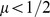). The inequality 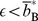 is equivalent to Eq. (38). Given Eq. (38), the equilibrium probability of Buy (i.e., 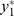) and that of Disc (i.e., 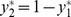) in the cooperative equilibrium, shown in Eq. (24), are derived by substituting Eq. (42) in
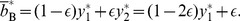
(44)To show the stability of the cooperative equilibrium, we first compare 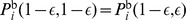, i.e., the payoff for Buy and that for Disc, and 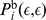, i.e., the payoff for NoBuy, in the cooperative equilibrium. NoBuy gains a smaller payoff than Buy and Disc if the coefficient of 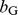 in Eq. (12) is positive in the cooperative equilibrium. The substitution of Eqs. (42) and (43) in Eq. (12) suggests that this condition is equivalent to
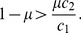
(45)
Equation (45) is equivalent to 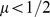, which we have assumed. Therefore, NoBuy gains a smaller payoff than Buy and Disc. Because 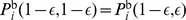 implies 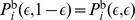, AntiDisc also gains a smaller payoff than Buy and Disc in the cooperative equilibrium.

Consider the invariant subspace of the strategy space where only Buy and Disc buyers (and C and D sellers) exist. The mixed strategy specified by 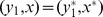 is a Nash equilibrium when the buyer's strategies are restricted to either Buy and Disc because 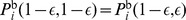 and 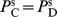. Because 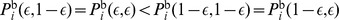, 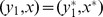 is a Nash equilibrium when all the four types of buyer's strategies are allowed.

The inequality 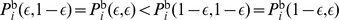 also assures that, under the replicator dynamics, the cooperative equilibrium is asymptotically stable against the introduction of an infinitesimal fraction of AntiDisc or NoBuy. Therefore, we are left to show the asymptotic stability of the cooperative equilibrium within the abovementioned two-dimensional subspace parametrized by 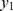 and 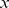. For the sake of the linear stability analysis, we take 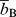 and 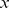 as the independent variables and linearize Eqs. (27) with 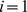 and (28) and substitute the one-to-one relationship between 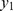 and 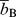 given by Eq. (4) in Eq. (27). The Jacobian in the equilibrium is given by
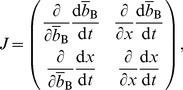
(46)where all the derivatives are evaluated at 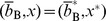, and
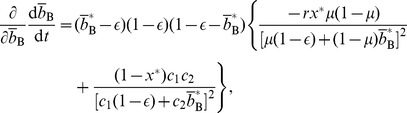
(47)

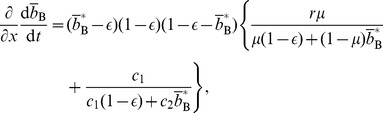
(48)

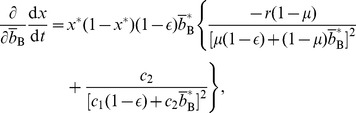
(49)

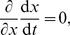
(50)The necessary and sufficient condition for the cooperative equilibrium to be stable under the replicator dynamics is given by 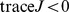 and 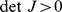. By substituting Eqs. (42) and (43) in Eq. (47), we obtain
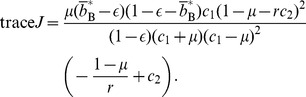
(51)Because the right-hand side of Eq. (49) is positive, we obtain
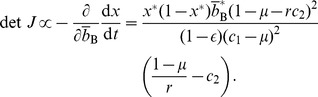
(52)
Equations (51) and (52) suggest that the cooperative equilibrium is stable if and only if
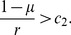
(53)
Equation (53) is satisfied for any 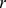 (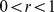) if 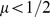. Therefore, the cooperative equilibrium is asymptotically stable under the replicator dynamics.

*Mixture of Disc and AntiDisc*: If Disc and AntiDisc are mixed in the equilibrium,
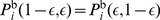
(54)holds true. Because 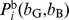 is linear in 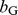 and 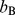, the coefficient of 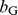 and that of 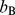 must be the same for Eq. (54) to be satisfied. If both coefficients are positive, the payoff for Buy is larger than that for Disc and AntiDisc in this equilibrium. If both coefficients are negative, the payoff for NoBuy is larger than that for Disc and AntiDisc in the equilibrium. In either case, the mixture of Disc and AntiDisc cannot be Nash.

*Mixture of Disc and NoBuy*: If Disc and NoBuy are mixed in the equilibrium, we obtain 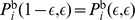 and 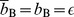. Therefore, the coefficient of 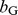 in Eq. (12), which we denote by 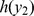 as a function of the density of Disc, is represented as
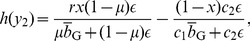
(55)where
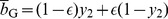
(56)must be equal to 0 in the equilibrium. From 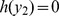 and 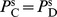, we obtain
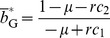
(57)and
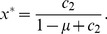
(58)If 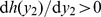 in the equilibrium, Disc (NoBuy) in a mixed strategy in which there are slightly more (less) probability of Disc than in the equilibrium 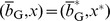 obtains a larger payoff than NoBuy (Disc). In this case, 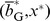 is not Nash because such a slightly modified mixed strategy of the buyer obtains a larger payoff than 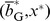. On the basis of the relationship 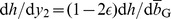, which is derived from Eq. (56), we rewrite 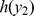 as 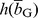 and examine 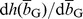 in the equilibrium (note that we assumed 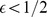). Using the fact that the right-hand side of Eq. (55) is equal to 0 when 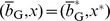, we obtain
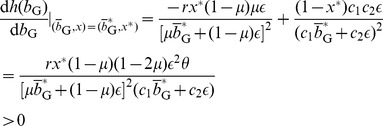
(59)if 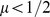. Therefore, the mixture of Disc and NoBuy is not Nash.

#### Mixture of three or four buyer's strategies

If three or four buyer's strategies are mixed in an equilibrium, their payoffs must be identical. Therefore, the coefficient of 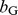 and that of 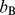 in Eq. (12) must be equal to 0, which requires 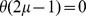. Because 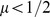, this relationship is not satisfied when the image scorer exists (i.e., 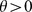).

When 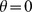, we obtain 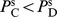 by substituting 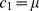 and 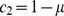 (derived from Eqs. (10) and (11)) in Eqs. (14) and (15). Therefore, 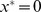 must hold in the equilibrium. In this situation, Eq. (12) is reduced to
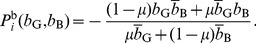
(60)
Equation (60) indicates that NoBuy's payoff is larger than Disc's and AntiDisc's payoffs, which are larger than Buy's payoff. Consequently, three or four buyers' strategies cannot be mixed in an equilibrium. Here is the end of the proof.

### Indifferent scoring

In a case with only indifferent scorers (i.e., 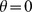), Eq. (26) is never satisfied (and Eq. (31) is not satisfied, either). Therefore, the uncooperative equilibrium is the only Nash equilibrium. This outcome is expected because, under indifferent scoring, the players perform the usual trust game [28]–[31].

### Image scoring

**Figure 2:**
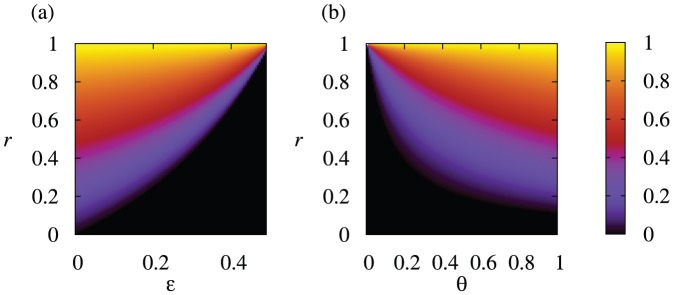
Probability of Buy 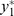 in the cooperative equilibrium in the limit 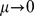. We set (a) 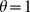 and (b) 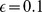.

When there are only image scorers (i.e., 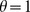), the cooperative equilibrium is realized in a wide parameter region because Eq. (26) with 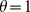 and 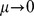 is reduced to 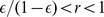; 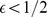 is the probability that the buyer misimplements the action. In the limit 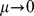, the equilibrium probability of Buy (i.e., 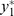) is plotted as a function of 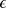 and 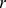 in Fig. 2 (a). In the parameter region in which 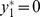 (black region in Fig. 2 (a)), the cooperative equilibrium does not exist. In the cooperative equilibrium, the fraction of Buy is large for a large 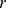 or a small 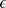. In particular, 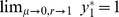 irrespective of the value of 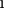. In the limit 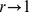, the trust game is a weak social dilemma such that the D seller's payoff is only infinitesimally larger than the C seller's payoff. The advantage of the D seller is offset by the B reputation that the D seller receives from just a small fraction of Disc buyers (i.e., 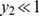). Even if a majority of buyers is nondiscriminative Buy, cooperation between buyers and sellers can be sustained by the reputation-regarding behavior of a small fraction of Disc.

For a general value of 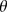, suppose that Eq. (26) is satisfied such that the cooperative equilibrium is Nash. For 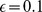 and 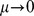, 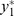 in the cooperative equilibrium is shown for various values of 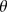 and 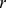 in Fig. 2 (b). 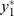 increases as 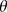 and 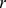 increase.

**Figure 3:**
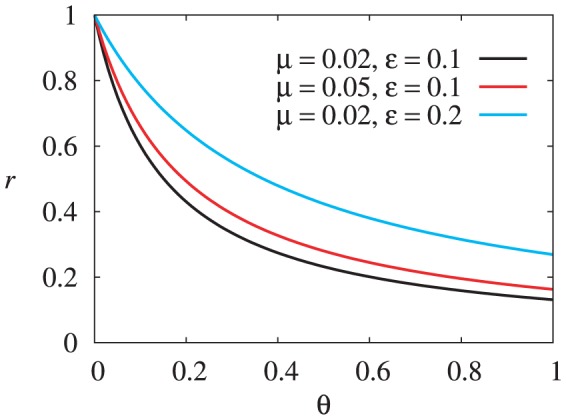
The threshold value of 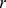 above which the cooperative equilibrium exists and is stable.

For some values of 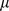 and 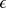, the critical line obtained from Eq. (26) is plotted in Fig. 3. The cooperative equilibrium exists and is stable above the critical line. Figure 3 indicates that cooperation based on the reputation mechanism occurs in a large parameter region, particularly for large values of 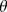 and 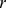. The critical line is rather insensitive to 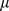 and relatively sensitive to 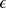. Nevertheless, cooperation is possible even for a large probability of the implementation error 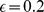.

### Attractive basin of the cooperative and uncooperative equilibria

The cooperative and uncooperative equilibria can coexist. A fuller understanding of the model requires a global analysis of the replicator dynamics to determine which of the two equilibria is more likely to be attained. In addition, periodic or chaotic attractors may exist when a population evolves. To exclude this possibility and examine the attractive basin of the two equilibria, we numerically run the standard two-population replicator dynamics [43]–[45] from various initial conditions to identify the limit set of the dynamics. For fixed parameter values, we assume that the initial condition is distributed according to the uniform density on the state space, i.e., 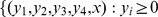 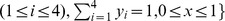. It should be noted that Eq. (5) is preserved under the replicator dynamics because the buyer's payoff depends on the buyer's strategy but is independent of whether the buyer is an indifferent or image scorer. We have implicitly assumed in Eqs. (27) and (28) that sellers and buyers have identical adaptation rates. However, the following results are qualitatively the same even if the two adaptation rates are different.

The replicator dynamics are four-dimensional, with three degrees of freedom derived from the buyer's population and one degree of freedom derived from the seller's population. Note that the selection occurs separately among buyers and sellers.

**Figure 4:**
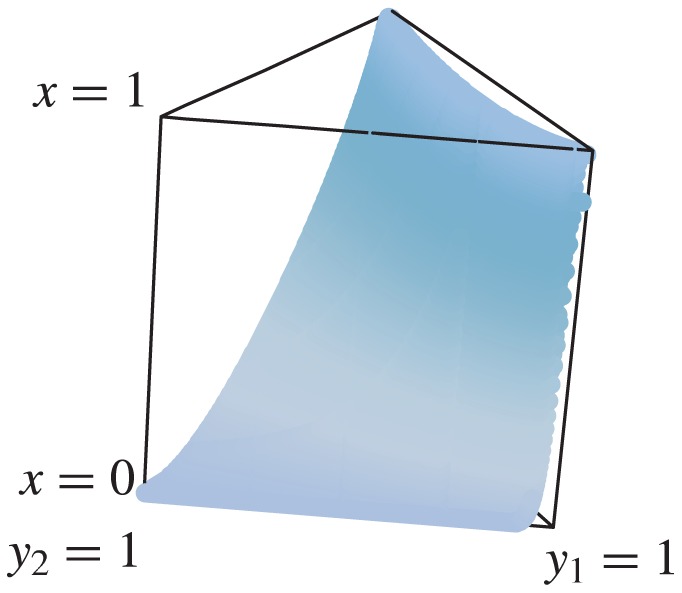
Boundary between the attractive basins of the cooperative and uncooperative equilibria. The points above the boundary are attracted to the cooperative equilibrium. We set 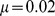, 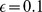, 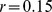, and 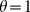.

For an expository purpose, we start with the system without AntiDisc. There are three strategies for buyers and two strategies for sellers. The replicator dynamics are three-dimensional, and the state space is a triangular prism defined by 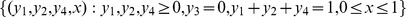. For 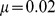, 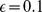, 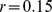, and 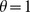, initial conditions located below the boundary shown in Fig. 4 are attracted to the uncooperative equilibrium. We confirmed that all the complementary regions in the interior of the triangular prism are attracted to the cooperative equilibrium. Our numerical simulations strongly suggest that there is no other limit set.

**Figure 5:**
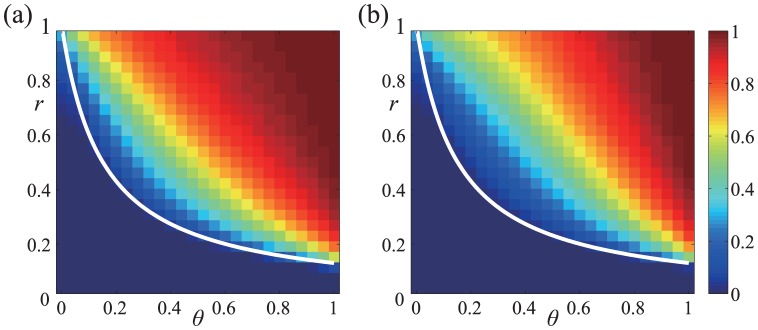
Relative volume of the attractive basin of the cooperative equilibrium. We set 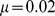 and 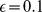. Initially, (a) three and (b) four buyer's strategies are distributed according to the uniform density.

To better quantify the possibility of cooperation, we measure the volume of the attractive basin of the cooperative equilibrium. The relative volume of the attractive basin in the triangular prism is shown in Fig. 5 (a) for 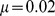, 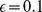, and various values of 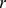 and 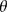. The critical line for the existence and stability of the cooperative equilibrium, implied by Eq. (26), is shown by the solid line. We find that the cooperative equilibrium is attractive for a substantial variety of initial conditions as 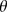 and 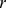 increase.

In the presence of four strategies for buyers, the relative volume of the attractive basin of the cooperative equilibrium in the state space is shown in Fig. 5 (b). The results are qualitatively the same as those shown in Fig. 5 (a). We confirmed that the rest of the state space belongs to the attractive basin of the uncooperative equilibrium. The introduction of AntiDisc does not inhibit the evolution of cooperation.

## Discussion

For the trust game, we have shown that cooperation between unacquainted buyers and sellers can be established under the image scoring norm (i.e., reputation mechanism). In the cooperative equilibrium, the population of buyers (i.e., investors) is a mixture of Buy (unconditional buyer) and Disc (discriminator that decides whether to buy depending on the seller's reputation). The majority of sellers (i.e., trustees) reciprocates the buyer's trust although the sellers are not expected to meet the same buyers again. It should be noted that not every buyer discriminates between good and bad sellers in the cooperative equilibrium. This feature is shared by some previous models of indirect reciprocity [14], [17], [19]. The probability of discriminators can be small depending on the parameter values. In addition, the buyer's reputation may be of little practical use for maintaining cooperative transactions. This claim is consistent with the previous theoretical result [6] and the empirical finding that reputation for a seller has a greater impact than that for a buyer [3], [5], [10].

Our model and results are distinct from previous ones obtained from the models using the symmetrized donation game, which we call indirect reciprocity games for now [8], [9], [11]–[16]. First, when cooperation prevails, a player with a good reputation is helped by unacquainted players in the indirect reciprocity games. In our model, a seller with a good reputation wins the trust, not explicit help, of unacquainted buyers. By reciprocating the buyer's trust, the seller obtains a relatively large momentary payoff and a good reputation. Then, a good reputation elicits trust and long-term cooperation from buyers.

A second difference is in the consequence of the image scoring. In the indirect reciprocity games, defection against a bad player is regarded to be bad under the image scoring. Therefore, the image scoring does not yield cooperation [13]–[16]. In contrast, in our model, a discriminative buyer (i.e., Disc) that defects against (i.e., does not buy from) a bad seller does not receive a bad reputation. This is because buyers do not own reputation scores by definition; the asymmetry of roles in the trust game allows the image scoring to support cooperation. Although a previous paper discussed this issue before [46], we analytically derived the conditions under which cooperation based on the image scoring occurs. It should be noted that the image scoring norm is simpler than the social norms required for cooperation in the indirect reciprocity games [13]–[16], [18], [19].

In other models, the mere reputation mechanism enables cooperation among unacquainted players in the trust game [9], [25] and other games [9], [24], [26], [27]. However, the component social dilemma game in these studies is essentially symmetric. Our model is inherently asymmetric such that a player is either a permanent buyer or permanent seller. As compared to a recent paper in which impacts of asymmetric roles in the trust game are numerically studied [46], we analytically established the conditions under which reputation-based cooperation occurs in the asymmetric trust game. In online marketplaces [10], the market for lemons [1], and presumably many other transaction scenes, some people may participate entirely or mostly as buyers and others as sellers.

The constructive role of reputations for the trustee (i.e., seller) in asymmetric interactions was formulated in a classic paper many years ago [6]. Our contribution to the understanding of this established mechanism is that we have clarified competition among different strategies using evolutionary game theory.

We have investigated a scenario in which indifferent scorers, which do not essentially score sellers, and image scorers coexist in a buyer's population. Remarkably, the fraction of image scorers needed for cooperation is not large; Eq. (26) indicates that the threshold fraction of image scorers is equal to 0.60 for 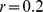 and 0.038 for 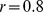 when 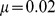 and 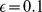. An alternative assumption for the behavior of indifferent scorers is that they do not alter sellers' scores rather than always give a good reputation to sellers. Analysis of this case is warranted for future work.

An important limitation of the present study is that the fraction of indifferent scorers and that of image scorers are invariant over time. In other words, indifferent and image scorers are assumed to receive the same payoff if the strategy (Buy, Disc, AntiDisc, or NoBuy) is the same. In fact, the image scoring may be more costly than the indifferent scoring because the image scorer has to know and report whether sellers cooperate or defect. Therefore, an image scorer may be tempted to turn into an indifferent scorer if the incentive to rate sellers is absent [2], [3], [10]. A similar cost is briefly mentioned in previous literature in the context of indirect reciprocity games [9], [47]. In our model, cooperation disappears if there are too many indifferent scorers. This result parallels with that for the indirect reciprocity games in which cooperation is not realized if there are too few observers in the one-shot game [11], [16]. In practice, rewarding image scorers and shutting down indifferent scorers' access to reputation information, for example, are means to circumvent the scoring cost [3]. We remark that the competition of social norms was analyzed in different models of indirect reciprocity [20]–[22].

In the context of asymmetric interaction between cleaner and client fishes, indirect reciprocity was investigated in an inherently asymmetric variant of the trust game with a binary internal state for the trustee [23]. Our model and results are distinct from theirs. In their model, a trustee reciprocates and attracts the investor when the trustee is in one particular state and exploits the investor by switching to the other state. The trustee does not steadily maintain a good reputation and uses the temporarily good reputation to exploit the investor. The authors acknowledge that their mechanism is different from the conventional concept of indirect reciprocity. In our model, the trustee (i.e., seller) steadily maintains a good reputation to invoke help from the investor (i.e., buyer). Our results suggest that, under appropriate conditions, the conventional indirect reciprocity may be established between cleaner and client fishes without resorting to the concept of the binary state. Finally, beyond the relevance to online transactions, our results provide a firm solution to the moral hazard problem that is represented by the trust game. Examples include offline markets [6] such as the market for lemons [1] and labor markets [29], [33]. Implementing a reputation mechanism only for the seller (i.e., investee) induces trust and cooperation between unacquainted individuals in the trust game. Good cooperative sellers and trustful buyers coevolve.
